# Peripheral human CD4^+^CD8^+^ T lymphocytes exhibit a memory phenotype and enhanced responses to IL-2, IL-7 and IL-15

**DOI:** 10.1038/s41598-017-11926-2

**Published:** 2017-09-14

**Authors:** Marie-Laure Clénet, François Gagnon, Ana Carmena Moratalla, Emilie C. Viel, Nathalie Arbour

**Affiliations:** Department of Neurosciences, Université de Montréal and CRCHUM, Montreal, QC H2X 0A9 Canada

## Abstract

CD4^+^CD8^+^ T lymphocytes account for 1–2% of circulating human T lymphocytes, but their frequency is augmented in several diseases. The phenotypic and functional properties of these T lymphocytes are still ill-defined. We performed an *ex vivo* characterization of CD4^+^CD8^+^ T lymphocytes from the blood of healthy individuals. We observed that CD4^+^CD8^+^ T lymphocytes exhibit several characteristics associated with memory T lymphocytes including the expression of chemokine receptors (e.g. CCR7, CXCR3, CCR6) and activation markers (e.g. CD57, CD95). Moreover, we showed that a greater proportion of CD4^+^CD8^+^ T lymphocytes have an enhanced capacity to produce cytokines (IFNγ, TNFα, IL-2, IL-4, IL-17A) and lytic enzymes (perforin, granzyme B) compared to CD4^+^ and/or CD8^+^ T lymphocytes. Finally, we assessed the impact of three key cytokines in T cell biology on these cells. We observed that IL-2, IL-7 and IL-15 triggered STAT5 phosphorylation in a greater proportion of CD4^+^CD8^+^ T lymphocytes compared to CD4 and CD8 counterparts. We demonstrate that CD4^+^CD8^+^ T lymphocytes from healthy donors exhibit a phenotypic profile associated with memory T lymphocytes, an increased capacity to produce cytokines and lytic enzymes, and a higher proportion of cells responding to key cytokines implicated in T cell survival, homeostasis and activation.

## Introduction

During thymic maturation, thymocytes expressing both CD4 and CD8 molecules develop into fully mature T lymphocytes carrying either CD4 or CD8. Subsequently, these mature naïve CD4^+^ or CD8^+^ T lymphocytes migrate to lymphoid organs where they can be efficiently activated in response to their cognate antigen presented by major histocompatibility complex molecules and appropriate co-stimulation. Although the commitment to mutually exclusive expression of CD4 or CD8 has been shown to be stringently regulated by transcription factors^1^, peripheral T lymphocytes expressing both CD4 and CD8 are detected in several species, including humans^1–5^. CD4^+^CD8^+^ T lymphocytes represent 1–2% of circulating human T lymphocytes^1^. However, numerous groups reported an augmented frequency of these cells in patients suffering from various disorders^1^ such as HIV^6^, hepatitis^7^, melanoma^8^, breast cancer^9^, rheumatoid arthritis^10^, and Chagas disease^11^. CD4^+^CD8^+^ T lymphocytes have been shown to produce pro-inflammatory cytokines and exert cytotoxicity especially in disease conditions^6, 8, 10, 12, 13^. Investigators have suggested that CD4^+^CD8^+^ T lymphocytes are highly activated cells exhibiting an effector memory phenotype^7, 14^. On the other hand, other studies have attributed regulatory properties to CD4^+^CD8^+^ T lymphocytes in animal models^15, 16^ and enhanced production of Th2 associated cytokines (interleukin-4 (IL-4) and IL-13) compared to single positive counterparts in human cancer^17^. Nevertheless, the phenotypic properties and functions of CD4^+^CD8^+^ T lymphocytes remain incompletely characterized.

The development, homeostasis, survival and activation of T lymphocytes are considerably shaped by the pleiotropic cytokines: IL-2, IL-7 and IL-15. Studies performed using animals deficient for any of the abovementioned cytokines have illustrated the non-overlapping and complementary impact of these cytokines on T cell biology^18^. Whereas IL-2 deficient mice have diminished number of regulatory T cells (Tregs)^19^, IL-15-deficient mice exhibit marked reductions in the numbers of memory CD8 T cells^20, 21^ and IL-7-deficient mice have a severe reduction in total T cell numbers^22^. These three cytokines share one receptor chain, the common gamma chain (CD132). As IL-2 and IL-15 share CD122 and CD132 signalling chains, they mediate similar functions. Nevertheless, IL-15 displays unique properties and targets a broader range of cells compared to IL-2^23^. IL-15 prevents the suppressive effect of Tregs on T cells^24^, whereas IL-2 is required to maintain these cells (CD4^+^CD25^+^)^19^. Additionally, IL-15 can inhibit IL-2-activation induced cell death of T cells^25^. IL-7 binds and signals via the CD127 (IL-7Rα) and CD132 chains^26^. IL-7 favours naïve and memory T lymphocyte survival via the up-regulation of anti-apoptotic proteins such as members of the Bcl-2 family^27^. Several groups have documented the variable responses of T cell subsets to these three key cytokines; whether peripheral CD4^+^CD8^+^ T lymphocytes respond differently to IL-2, IL-7 and IL-15 compared to other T cell subsets has not been previously investigated. Given the growing interest in modulating the levels of these cytokines for therapeutic interventions in multiple disorders^26^, a better understanding of the impact of these cytokines on all human T cell subsets including CD4^+^CD8^+^ T cells is deemed highly relevant.

In this report, we compared peripheral CD4^+^CD8^+^ T lymphocytes to CD4^+^ and CD8^+^ T lymphocyte subsets for multiple parameters including phenotypic characterization, cytokine and lytic enzyme production, and responses to IL-2, IL-7 and IL-15. We provide evidences that CD4^+^CD8^+^ T lymphocytes exhibit a memory phenotype and an enhanced capacity to produce cytokines and lytic enzymes compared to CD4^+^ and CD8^+^ T cells. Moreover, IL-2, IL-7 and IL-15 can trigger STAT5 phosphorylation in a greater proportion of CD4^+^CD8^+^ T lymphocytes compared to other T cell subsets supporting the unique features of these cells.

## Results

### Peripheral CD4^+^CD8^+^ T lymphocytes display characteristics of memory T lymphocytes

**Figure 1:**
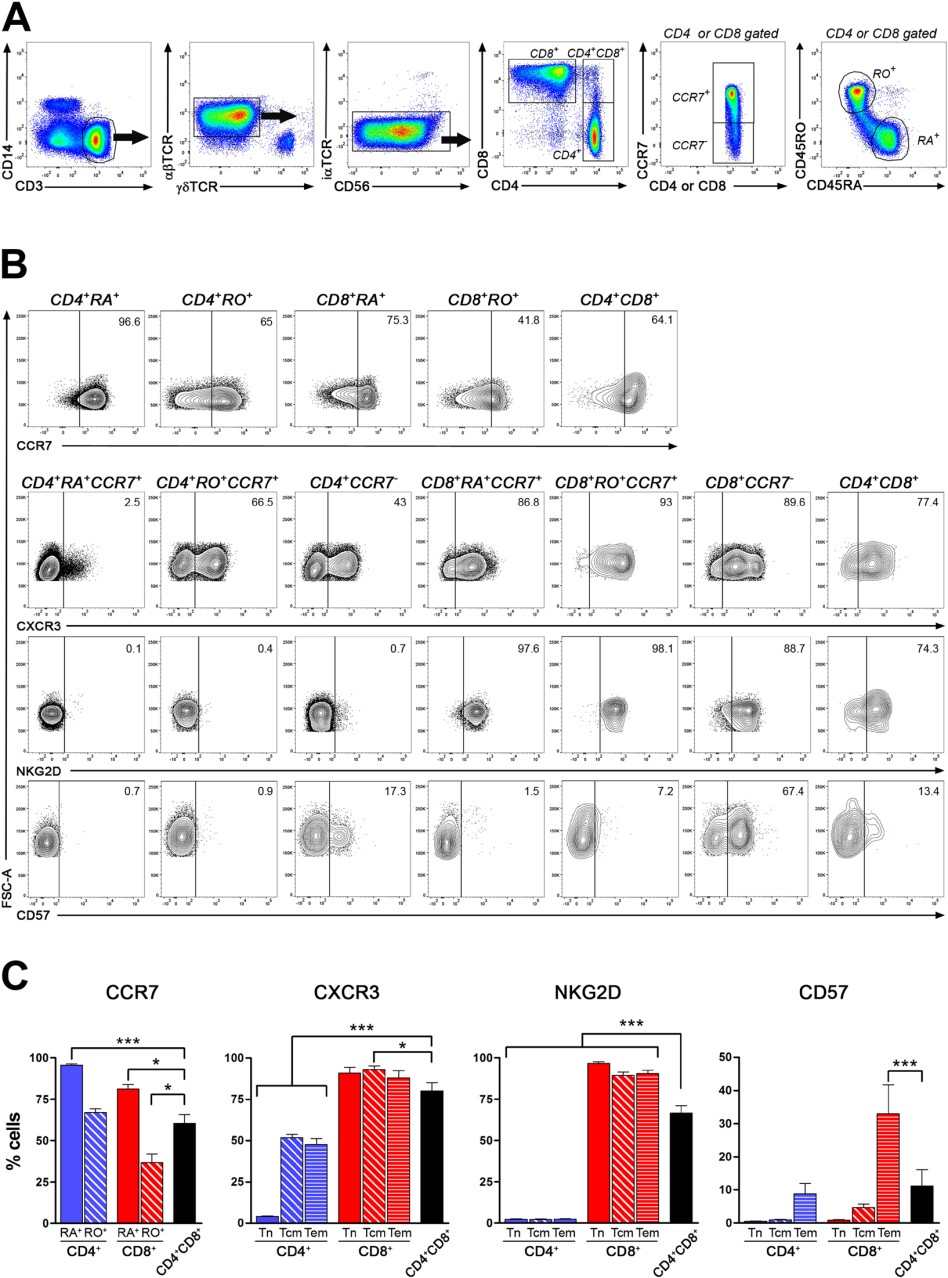
CD4^+^CD8^+^ T lymphocytes represent a distinct subset from CD4 and CD8 T lymphocytes. *Ex vivo* PBMCs were stained for multiple surface markers to characterize CD4^+^CD8^+^ T lymphocytes. (**A**) Gating strategy from one donor is illustrated; cells debris, doublets and dead cells were excluded from analysis. CD14^−^CD3^+^ T lymphocytes were selected and then αβTCR^+^ cells were gated while γδTCR^+^ (second panel) and CD56^+^ invariant TCR (Vα24 Jα18)^+^ cells (third panel) were excluded. T lymphocytes expressing either CD4^+^, CD8^+^ or CD4^+^CD8^+^ were analysed. For CD4^+^ and CD8^+^ T cells, CCR7^+^ cells were further subdivided into CD45RA^+^ or CD45RO^+^ cells. (**B**) Representative dot plots of PBMCs gated on either CD4^+^CD45RA^+^, CD4^+^CD45RO^+^, CD8^+^CD45RA^+^, CD8^+^ CD45RO^+^ or CD4^+^CD8^+^ T cell subsets illustrating CCR7 expression (top row). For the other panels, expression of CXCR3 (second row), NKG2D (third row) and CD57 (forth row) are depicted gated on CD4^+^CD45RA^+^CCR7^+^ (T naïve, Tn), CD4^+^CD45RO^+^CCR7^+^ (T central memory, Tcm), CD4^+^CCR7^−^ (T effector memory, Tem), CD8^+^CD45RA^+^CCR7^+^(Tn), CD8^+^CD45RO^+^CCR7^+^ (Tcm), CD8^+^CCR7^−^ (Tem) or CD4^+^CD8^+^ T cells. Quadrants were drawn according to isotype and FMO controls. (**C**) For each cell subset, percentage of cells expressing CCR7 (n = 13), CXCR3 (n = 10), NKG2D (n = 8), and CD57 (n = 8) are shown: mean ± SEM. ANOVA followed by Dunnett’s as *post hoc* test comparing CD4^+^CD8^+^ T cell subset to all other subsets * p < 0.05, ***p < 0.001.

Previous studies suggested that CD4^+^CD8^+^ T lymphocytes share attributes of activated effector T cells^7, 14^. However, whether peripheral human CD4^+^CD8^+^ T lymphocytes carry molecules linked to specific subsets of T lymphocytes remains unclear. We compared the expression of markers associated with the activation state, migratory properties and regulatory functions of T lymphocytes in *ex vivo* PBMCs obtained from healthy donors. Representative data from one donor are illustrated in Fig. 1A,B, and results compiled from multiple donors are shown in Fig. 1C. CD4^+^CD8^+^ T lymphocytes represent a subpopulation distinct from CD4^+^ and CD8^+^ single positive T lymphocytes (Fig. 1A), accounting for 1.0 ± 0.2% of all CD3^+^αβTCR^+^ T lymphocytes. Most CD4^+^CD8^+^ T lymphocytes expressed the heterodimer CD8αβ as previously reported^1, 28^, but these cells did not express γδTCR nor the invariant chain Vα24 Jα18 (data not shown). The proportion of CD4^+^CD8^+^ T lymphocytes (60.4 ± 5.3%) expressing CCR7, a molecule involved in the recirculation of lymphocytes to lymph nodes^29^, was significantly lower compared to CD45RA^+^CD4 (95.6 ± 0.8%) and CD45RA^+^CD8 (81.1 ± 2.7%) T lymphocytes; but higher than CD45RO^+^CD8 T lymphocytes (36.6 ± 5.4%) and similar to CD45RO^+^CD4 T lymphocytes (66.9 ± 2.2%) (Fig. 1B upper row and Fig. 1C). Our results suggest that more than half of CD4^+^CD8^+^ T cells could be prone to recirculate in the periphery as they bear CCR7. Thereafter, we used CCR7 expression to discriminate naïve (Tn: CD45RA^+^CCR7^+^), central memory (Tcm: CD45RO^+^CCR7^+^) and effector memory (Tem: CCR7^−^) T lymphocyte subpopulations.

We looked for the presence of CXCR3, a key chemokine receptor that is greatly expressed by effector T lymphocytes^30^ and previously shown to be detected on a greater portion of CD4^+^CD8^+^ T lymphocytes compared to CD4 T cells^7^. Indeed, we observed that the percentage of CD4^+^CD8^+^ T lymphocytes (80.2 ± 4.9%) expressing CXCR3 was significantly greater than CD4 T lymphocyte subsets (Tn: 4.2 ± 0.4%; Tcm: 51.7 ± 1.9%; Tem: 48.0 ± 3.5%) but slightly lower than CD8 T lymphocyte subsets (Tn: 90.8 ± 3.7%; Tcm: 93.0 ± 2.4%; Tem: 88.0 ± 4.4%) (Fig. 1B second row and Fig. 1C). NKG2D is a co-activating receptor present on most human CD8 T lymphocytes but only a few CD4 T lymphocytes^31^. We observed that a significantly greater proportion of CD4^+^CD8^+^ T lymphocytes expressed NKG2D (66.5 ± 4.5%) compared to CD4 T lymphocyte subsets (Tn: 2.2 ± 0.5%; Tcm: 2.1 ± 0.5%; Tem: 2.3 ± 0.4%) but this percentage was significantly lower than CD8 T lymphocytes (Tn: 96.7 ± 0.9%; Tcm: 89.2 ± 2%; Tem: 90.4 ± 2.0%) (Fig. 1B third row and Fig. 1C). We also assessed the expression of CD57, a marker associated with terminally differentiated senescent T lymphocytes^32^. A greater proportion of CD4^+^CD8^+^ T lymphocytes (11.2 ± 4.9%) carried CD57, compared to both naïve and central memory CD4 (Tn: 0.5 ± 0.1%; Tcm: 0.9 ± 0.2%); however, this percentage was significantly lower than what was detected in effector memory CD8 T lymphocytes (Tem: 33.0 ± 8.8%) (Fig. 1B forth row and Fig. 1C). Overall, our phenotypic characterization supports the notion that CD4^+^CD8^+^ T lymphocytes exhibit phenotypic properties reminiscent of memory T cells including markers (NKG2D, CXCR3) more prevalent on CD8 T lymphocytes.

### CD4^+^CD8^+^ T lymphocytes constitute a diversified population of memory T lymphocytes

**Figure 2:**
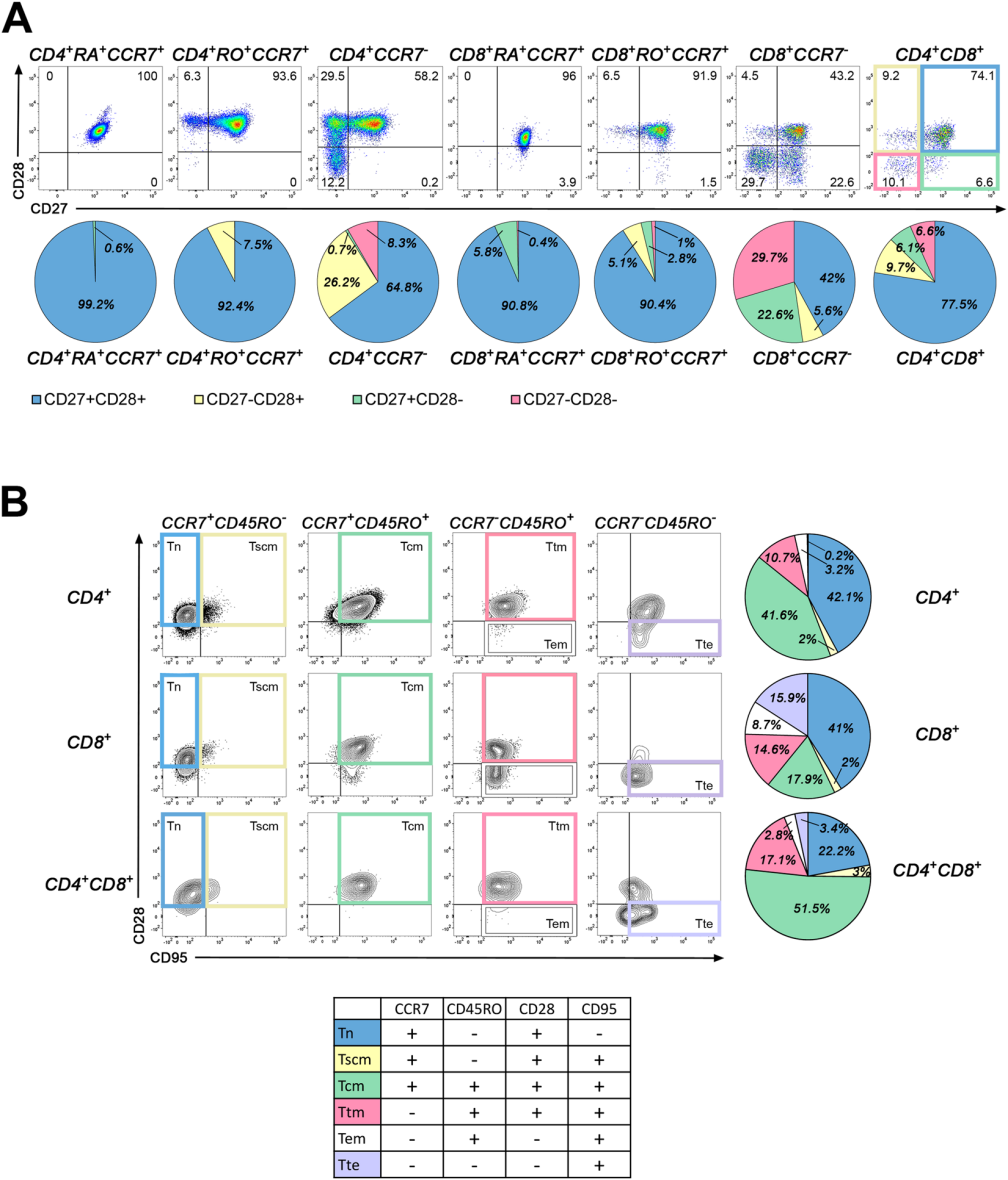
CD4^+^CD8^+^ T lymphocytes exhibit properties of memory T lymphocytes. Markers associated with different maturation stages were assessed on T cell subsets in *ex vivo* PBMCs. (**A**) Cells were stained for CD3, CD14, CD4, CD8, CD45RA, CD45RO, CCR7, CD27 and CD28. Representative dot plots from one donor gated on naïve (CD45RA^+^CCR7^+^), central memory (CD45RO^+^CCR7^+^) and effector memory (CCR7^−^) CD4 and CD8 T lymphocytes and CD4^+^CD8^+^ T lymphocytes illustrating CD27 and CD28 expression are depicted. For each cell subset, a pie chart representing the percentage of cells CD27^+^CD28^+^ (blue), CD27^−^CD28^+^(yellow), CD27^+^CD28^−^(green) and CD27^−^CD28^−^ (pink) obtained from 6 donors is shown. (**B**) PBMCs were stained for CD3, CD14, CD4, CD8, CD45RO, CCR7, CD28 and CD95. Representative contour plots from one donor gated on naïve (Tn: CCR7^+^CD45RO^−^CD28^+^CD95^−^, blue), stem cell memory (Tscm: CCR7^+^CD45RO^−^CD28^+^CD95^+^, yellow), central memory (Tcm: CCR7^+^CD45RO^+^CD28^+^CD95^+^, green), transitional memory (Ttm: CCR7^−^CD45RO^+^CD28^+^CD95^+^, pink), effector memory (Tem: CCR7^−^CD45RO^+^CD28^−^CD95^+^, white) and terminal effector (Tte: CCR7^−^CD45RO^−^CD28^−^CD95^+^, lavender) CD4, CD8 and CD4^+^CD8^+^ T lymphocytes. Percentage of each cell subset detected in CD4, CD8 and CD4^+^CD8^+^ T lymphocytes pooled from 6 donors are depicted in pie charts. Friedman test followed by Dunn’s multiple comparison test comparing CD4^+^CD8^+^ vs. CD4^+^ or CD8^+^ *p < 0.05 for the percentage of Tn (blue), CD4^+^CD8^+^ vs. CD8^+^ **p < 0.01 for the percentage ﻿of Tcm (green).

The activation and differentiation of naïve human CD4 and CD8 T lymphocytes into different memory subsets are correlated with the expression of specific surface markers^33^; to further characterize CD4^+^CD8^+^ T lymphocytes, we assessed several of these markers. Both CD27 and CD28 can provide co-stimulatory signals to T lymphocytes upon their encounter with antigen presenting cells expressing the cognate ligands, CD70 and CD80/CD86, respectively^34^. The loss of CD27 and/or CD28 is associated with an advanced stage of T cell differentiation characterized by desensitization to these stimulatory signals^33^. As expected, the vast majority of naïve CD4 (CD4^+^RA^+^CCR7^+^: 99.2 ± 0.4%) and CD8 (CD8^+^RA^+^CCR7^+^: 90.8 ± 2.5%) T lymphocytes were positive for both markers (Fig. 2A). Whereas less than 10% of central memory (CD45RO^+^CCR7^+^) CD4 and CD8 T cells lost CD27, CD28 or both markers, these proportions reached 35% and 58% for effector memory (CCR7^−^) CD4 and CD8 T cells respectively (Fig. 2A). Notably, the proportion of CD4^+^CD8^+^ T lymphocytes not carrying CD27, CD28 or both was 22.4%, which was higher than central memory but lower than effector memory CD4 and CD8 T lymphocytes (Fig. 2A).

Based on CCR7, CD45RO, CD28 and CD95 surface expression, it is possible to discriminate six differentiation stages of human T cells: naïve (Tn), stem cell memory (Tscm), central memory (Tcm), transitional memory (Ttm), effector memory (Tem) and terminal effector (Tte)^33^. We observed that CD4^+^CD8^+^ T lymphocytes contained a smaller proportion (22.2 ± 6%) of Tn cells (CCR7^+^CD45RO^−^CD28^+^CD95^−^, blue) compared to CD4 (42.1% ± 7.4%) and CD8 (41 ± 7.1%) T cells (Fig. 2B). Tscm (CCR7^+^CD45RO^−^CD28^+^CD95^+^, yellow) represented a small fraction (2–3%) of CD4, CD8 or CD4^+^CD8^+^ T cells. In contrast, about half (51.5 ± 4.5%) of CD4^+^CD8^+^ T cells exhibited the Tcm profile (CCR7^+^CD45RO^+^CD28^+^CD95^+^, green) whereas only 17.9% of CD8 and 41.6% of CD4 T cells expressed this profile. Notably, CD4^+^CD8^+^ T cells included a greater proportion (17.1 ± 3.4%) of Ttm (CCR7^−^CD45RO^+^CD28^+^CD95^+^, pink) than CD4 (10.7 ± 2.3%) and CD8 (14.6 ± 2.3%) T cells, whereas the percentage (2.8 ± 1.1%) of Tem (CCR7^−^CD45RO^+^CD28^−^CD95^+^, white) was comparable to CD4 (3.2 ± 2.2%) and lower than CD8 (8.7 ± 2.9%) T cells. Finally, the proportion of Tte (CCR7^−^CD45RO^−^CD28^−^CD95^+^, lavender) in CD4^+^CD8^+^ T cells (3.4 ± 2%) was higher than in CD4 (0.2 ± 0.2%) but lower than in CD8 T cells (15.9 ± 6.3%). Collectively, our results suggest that CD4^+^CD8^+^ T lymphocytes constitute a diversified population predominantly composed of subsets of memory T lymphocytes; 75% of these cells exhibited a Tcm, Ttm, Tem or Tte phenotype.

### CD4^+^CD8^+^ T lymphocytes exhibit enhanced cytokine and lytic enzyme production

**Figure 3:**
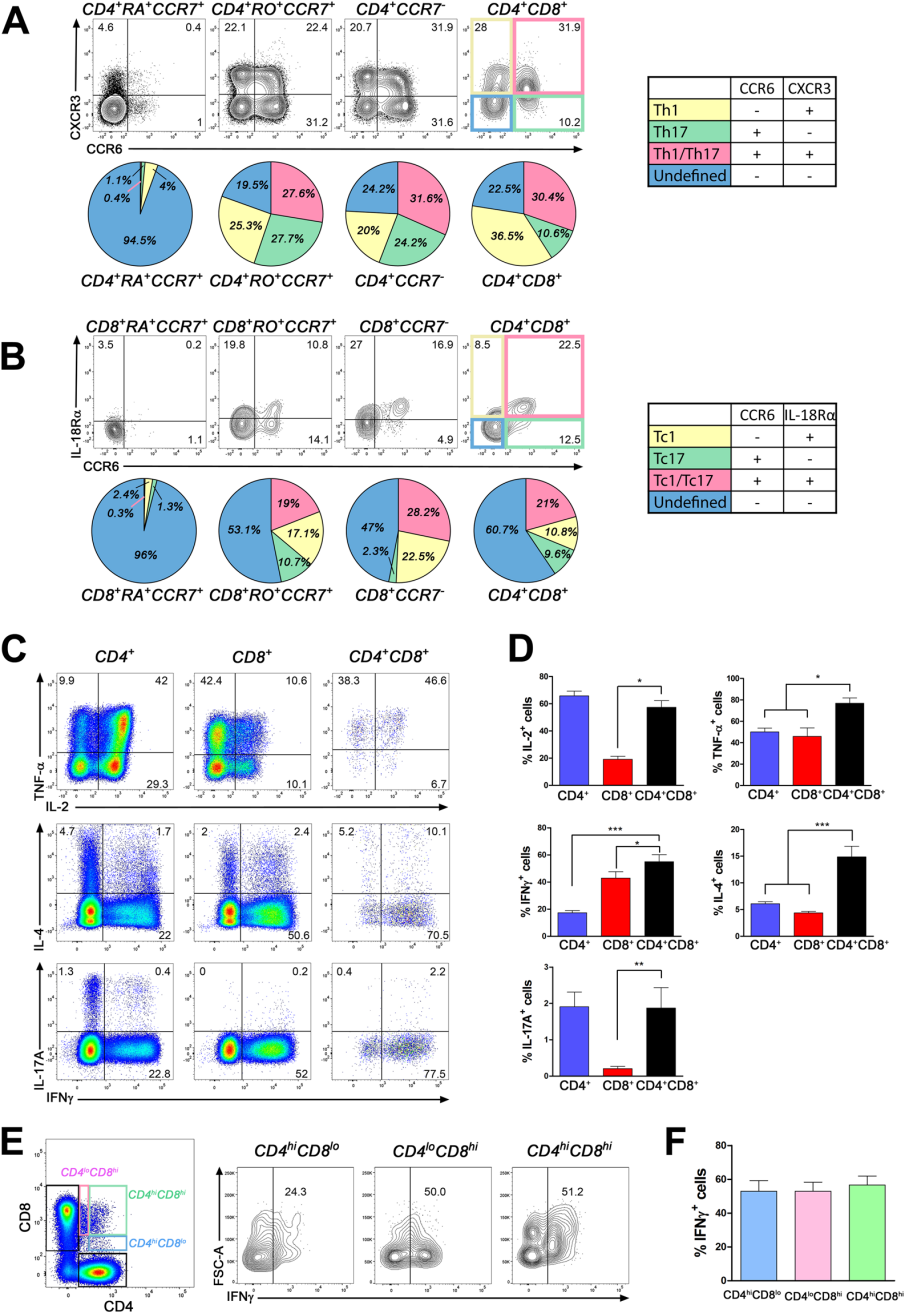
CD4^+^CD8^+^ T lymphocytes show enhanced cytokine production. (**A**) PBMCs were stained for CD3, CD14, CD4, CD8, CD45RA, CD45RO, CCR7, CCR6 and CXCR3. Contour plots from one donor gated on CD45RA^+^CCR7^+^, CD45RO^+^CCR7^+^ and CCR7^−^ CD4 and CD4^+^CD8^+^ T lymphocytes illustrating CCR6 and CXCR3 expression are depicted. Pie charts representing percentages of cells with Th1 (CCR6^−^CXCR3^+^, yellow), Th17 (CCR6^+^CXCR3^−^, green), Th1/Th17 (CCR6^+^CXCR3^+^, pink) and undefined profile (CCR6^−^CXCR3^−^, blue) (n = 6) are shown underneath each plot. Friedman test followed by Dunn’s multiple comparison test: CD4^+^CD8^+^ vs. CD4^+^CD45RA^+^CCR7^+^ *p < 0.05 for CCR6^+^CXCR3^+^ and CCR6^−^CXCR3^+^ cells. (**B**) PBMCs were stained for CD3, CD14, CD4, CD8, CD45RA, CD45RO, CCR7, CCR6 and IL-18Rα. Contour plots from one donor gated on CD45RA^+^CCR7^+^, CD45RO^+^CCR7^+^ and CCR7^−^ CD8 and CD4^+^CD8^+^ T lymphocytes illustrating CCR6 and IL-18Rα expression are shown. Percentages of cells with Tc1 (CCR6^−^IL-18Rα^+^, yellow), Tc17 (CCR6^+^IL-18Rα^−^, green), Tc1/Tc17 (CCR6^+^IL-18Rα^+^, pink) and undefined profile (CCR6^−^IL-18Rα^−^, blue) (n = 9) are depicted as pie charts underneath each contour plot. ANOVA followed by Bonferroni test comparing CD4^+^CD8^+^ to CD8^+^CD45RA^+^CCR7^+^ *p < 0.05 for CCR6^−^IL-18Rα^+^ and CCR6^+^IL-18Rα^+^ cells; ***p < 0.001 for CCR6^+^IL-18Rα^−^. Comparing CD4^+^CD8^+^ to CD8^+^CCR7^−^ **p < 0.01 for CCR6^−^IL-18Rα^+^ and CCR6^+^IL-18Rα^−^ cells. (**C**) PBMCs treated with PMA+ ionomycin + BFA for 5 h were stained for CD3, CD4, CD8, IFNγ, IL-4, IL-2, IL-17A and TNFα. Dot plots from one donor gated on CD4^+^, CD8^+^ and CD4^+^CD8^+^ T lymphocytes illustrate detection of IL-2 (upper row), TNFα (upper row), IFNγ (middle and bottom rows), IL-4 (middle row) and IL-17A (bottom row). (**D**) Percentages of CD4^+^, CD8^+^ and CD4^+^CD8^+^ T lymphocytes expressing IL-2 (n = 6), TNFα (n = 6), IFNγ (n = 13), IL-4 (n = 10) and IL-17A (n = 10) are depicted as mean ± SEM. Friedman test followed by Dunn’s multiple comparison test: CD4^+^CD8^+^ vs. CD4^+^ or CD8^+^ *p < 0.05 or **p < 0.01 for IL-2, TNFα, or IL-17A. ANOVA followed by Dunnett’s as *post hoc* test comparing CD4^+^CD8^+^ T cells to CD4^+^ or CD8^+^ subsets * p < 0.05, ***p < 0.001 for IFNγ or IL-4. (**E**) IFNγ production by CD4^hi^CD8^lo^ (blue), CD4^lo^CD8^hi^ (pink) and CD4^hi^CD8^hi^ (green) subsets. One representative dot plot for subset gating and contour plots for IFNγ production from one donor are shown. (**F**) Percentages of CD4^hi^CD8^lo^, CD4^lo^CD8^hi^ and CD4^hi^CD8^hi^ subsets expressing IFNγ (n = 13).

As most CD4^+^CD8^+^ T lymphocytes show a memory T cell phenotype, we investigated whether these cells exhibit properties associated with specific T cell polarization states. We assessed the *ex vivo* expression of chemokine receptors, CCR6 and CXCR3, which have been used to identify human Th1 (CCR6^−^CXCR3^+^, yellow), Th17 (CCR6^+^CXCR3^−^, green) and Th1/Th17 (CCR6^+^CXCR3^+^, pink) by others^35^. As expected very few (5%) naïve CD4 T cells had detectable levels of these chemokine receptors, but over 75% of central memory (CD45RO^+^CCR7^+^) and effector memory (CCR7^−^) CD4 T cells expressed either one or both receptors (Fig. 3A) in similar percentages. CD4^+^CD8^+^ T lymphocytes exhibited very similar proportion of Th1/Th17, a slightly elevated proportion of Th1 (CCR6^−^CXCR3^+^) and a marginally lower fraction of Th17 cells compared to CD4 T cell memory subsets (Fig. 3A).

Given that CD4^+^CD8^+^ T lymphocytes display characteristics of memory CD8 T lymphocytes, we also assessed the expression of IL-18Rα and CCR6 which have been associated with Tc1, Tc17 and Tc1/Tc17 subsets^36^. Similar to CD4 T cells, very few (4%) naïve CD8 T cells expressed these molecules (Fig. 3B). CD4^+^CD8^+^ T lymphocytes contained proportions of Tc1 (IL18Rα^+^CCR6^−^ yellow; 10.8%), Tc17 (IL18Rα^−^CCR6^+^ green; 9.6%) and Tc1/Tc17 (IL18Rα^+^CCR6^+^ pink; 21%) that were similar to central memory CD8 T cells (CD45RO^+^CCR7^+^). In contrast, the proportion of Tc1 (yellow) was significantly lower whereas the percentage of Tc17 cells (green) was significantly greater in CD4^+^CD8^+^ T lymphocytes compared to the effector memory CD8 T cells (CCR7^−^) (*p < 0.05) (Fig. 3B).

Activated T lymphocytes can be classified according to their production of specific effector molecules. We assessed whether CD4^+^CD8^+^ T lymphocytes have the capacity to produce cytokines associated with effector functions and/or Th1/Tc1, Th2/Tc2 or Th17/Tc17 subsets. Total PBMCs were activated during 5 h with PMA + ionomycin in the presence of BFA and then stained for interferon-gamma (IFNγ), IL-2, IL-4, IL-17A and tumour necrosis factor-α (TNFα). A greater percentage of CD4^+^CD8^+^ T lymphocytes (57.3 ± 4.9%) produced IL-2 compared to CD8 (19.1 ± 2.2%; *p < 0.05) T cells but this result was similar to CD4 T cells (65.8 ± 3.4%) (Fig. 3C upper row and Fig. 3D). TNFα was produced by a greater proportion of CD4^+^CD8^+^ T lymphocytes (76.8 ± 5%; *p < 0.05) compared to both CD4 (50.1 ± 3.5%) and CD8 (45.9 ± 7.9%) T cell subsets (Fig. 3C upper row and Fig. 3D). The Th1/Tc1 cytokine IFNγ was produced by a greater proportion of CD4^+^CD8^+^ (54.9 ± 5.2%) T lymphocytes than CD4 (17.3 ± 1.6%; compared to CD4^+^CD8^+^ T cells ***p < 0.001) and CD8 (42.9 ± 4.7%; compared to CD4^+^CD8^+^ T cells *p < 0.05) T cells (Fig. 3C middle and bottom rows and Fig. 3D). A higher percentage of CD4^+^CD8^+^ T lymphocytes (14.8 ± 2%) produced IL-4, the Th2/Tc2 associated cytokine, compared to CD4 (6.1 ± 0.4%) and CD8 (4.4 + 0.3%) (***p < 0.001 compared to CD4^+^CD8^+^) T cell subsets (Fig. 3C middle row and Fig. 3D). We detected similar proportions of CD4^+^CD8^+^ (1.9 ± 0.6%) and CD4 T cells (1.9 ± 0.4%) producing IL-17A, the Th17/Tc17 signature cytokine, but a significantly reduced percentage of CD8 T lymphocytes were positive for this cytokine (0.2 ± 0.1%; **p < 0.01 compared to CD4^+^CD8^+^) (Fig. 3C,D). CD4^+^CD8^+^ T cells can be divided into smaller subsets based on the different expression levels of CD4 and CD8^37, 38^. We compared the production of IFNγ by CD4^hi^CD8^lo^, CD4^lo^CD8^hi^ and CD4^hi^CD8^hi^ subsets (Fig. 3E,F). Although the proportion of cells expressing IFNγ varied between these subsets within one donor’s sample (Fig. 3E), overall, the percentages pooled from 13 donors were similar between all subsets (Fig. 3F).

**Figure 4:**
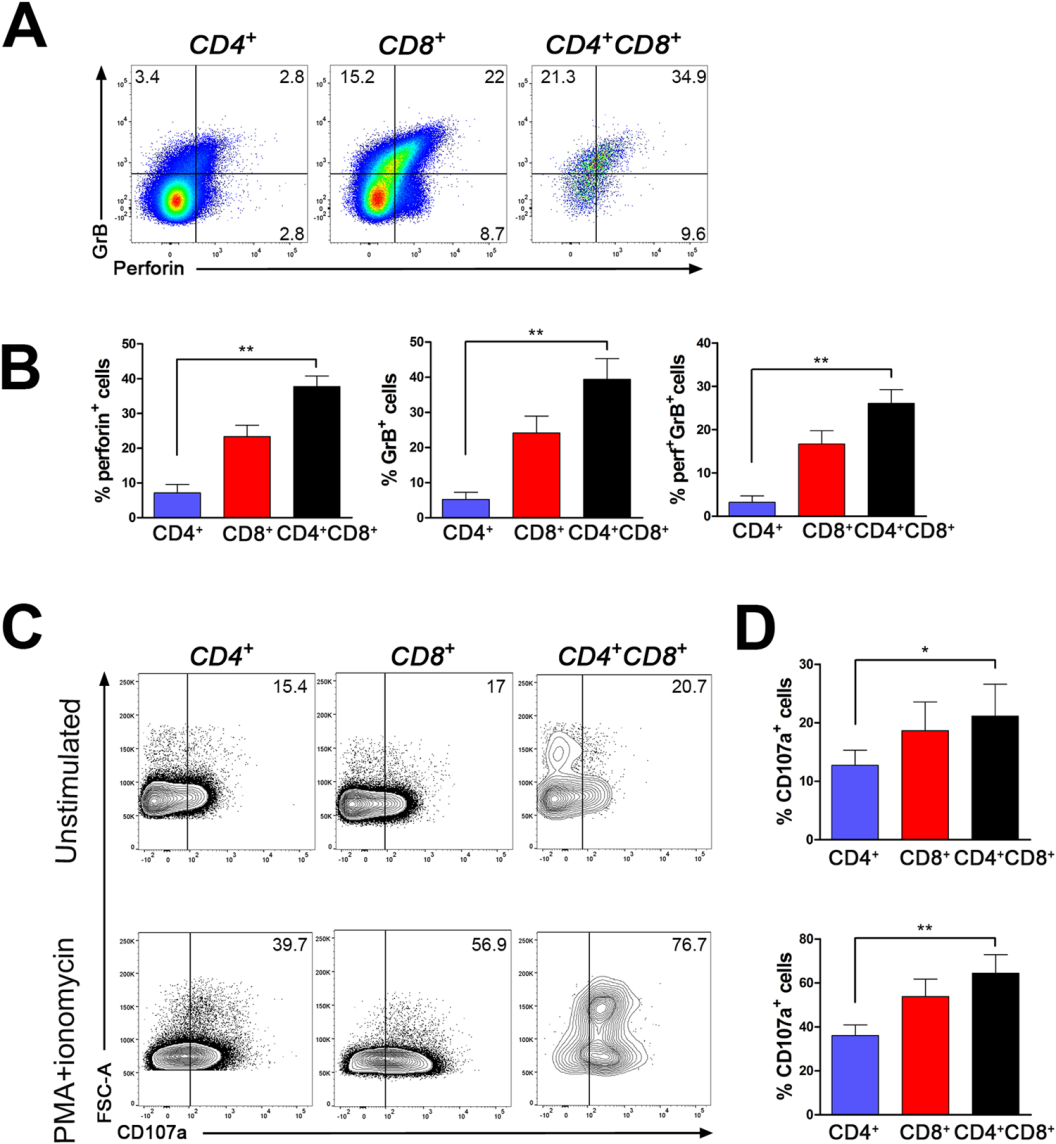
CD4^+^CD8^+^ T lymphocytes show enhanced lytic enzyme production. (**A**) PBMCs were treated with PMA+ ionomycin + BFA for 5 h and then stained for CD3, CD4, CD8, perforin and granzyme B (GrB). Representative dot plots from one donor gated on CD4^+^, CD8^+^ and CD4^+^CD8^+^ T lymphocytes illustrate detection of GrB and perforin. (**B**) Percentages of CD4^+^, CD8^+^ and CD4^+^CD8^+^ T lymphocytes expressing perforin, GrB or both perforin and GrB are depicted for 6 donors: mean ± SEM. Friedman test followed by Dunn’s multiple comparison test: CD4^+^CD8^+^ vs. CD4^+^ **p < 0.01 for the percentage of GrB^+^, Perforin^+^ or GrB^+^Perforin^+^. (**C**) PBMCs were incubated for 5 h with monensin and αCD107a in the absence or presence of PMA and ionomycin prior to being stained for CD3, CD4, and CD8. Representative dot plots from one donor gated on CD4^+^, CD8^+^ and CD4^+^CD8^+^ T lymphocytes illustrate CD107a detection in unstimulated or PMA + ionomycin stimulated cells. (**D**) Percentages of CD4^+^, CD8^+^ and CD4^+^CD8^+^ T lymphocytes expressing CD107a are depicted for 4 donors: mean ± SEM. Friedman test followed by Dunn’s multiple comparison test: CD4^+^CD8^+^ vs. CD4^+^ *p < 0.05 or **p < 0.01 for the percentage of CD107a^+^ cells.

Thereafter, we assessed the capacity of CD4^+^CD8^+^ T lymphocytes to produce lytic enzymes. Notably, CD4^+^CD8^+^ T lymphocytes contained the greatest proportion of cells carrying lytic enzymes (perforin and granzyme B) (26.1 ± 3.2%) compared to CD8 (16.7 ± 3%) and CD4 (3.2 ± 1.4%) T cells (Fig. 4A,B). As expected, a greater proportion of CD8 T cells expressed lytic enzymes compared to CD4 counterparts. We also measured the capacity of CD4^+^CD8^+^ T lymphocytes to degranulate. Total PBMCs were activated or not with PMA + ionomycin during 5 h in the presence of monensin. The proportion of CD4^+^CD8^+^ T lymphocytes expressing surface CD107a was augmented compared to CD4 T cells under both unstimulated and stimulated conditions but was similar to CD8 T cells (Fig. 4C,D).

Overall, our results demonstrate that CD4^+^CD8^+^ T lymphocytes represent a heterogeneous population of activated/memory T cells; this population has a higher proportion of cells with the capacity to produce effector molecules (IFNγ, TNFα, GrB, perforin) associated to the Th1/Tc1 profile. Moreover, CD4^+^CD8^+^ T lymphocytes display similarities to effector and central memory CD4 T cells (e.g. CCR6, CXCR3) and effector and central memory CD8 T lymphocytes (NKG2D, IL-18Rα).

### A greater proportion of CD4^+^CD8^+^ T lymphocytes responds to IL-2, IL-15 and IL-7

**Figure 5:**
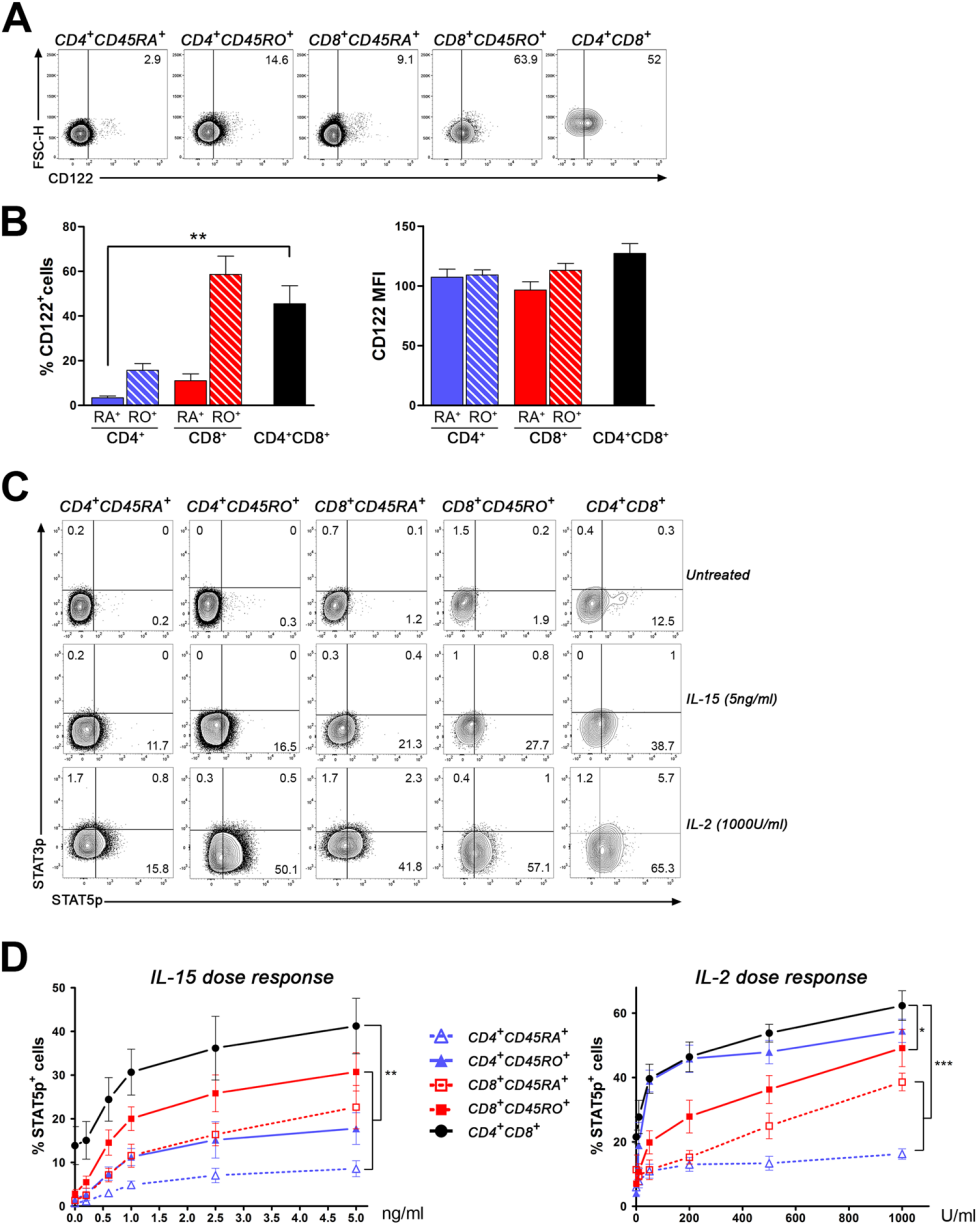
A greater proportion of CD4^+^CD8^+^ T lymphocytes respond to IL-15 and IL-2 compared to other T cell subsets. CD4^+^CD8^+^ T lymphocytes were compared to other T cell subsets for their expression of CD122, the receptor chain shared by IL-2 and IL-15, as well as their capacity to respond to these cytokines. (**A**) *Ex vivo* PBMCs were stained for CD3, CD14, CD4, CD8, CD45RA, CD45RO, and CD122. Representative contour plots of PBMCs from one donor gated on CD4^+^CD45RA^+^, CD4^+^CD45RO^+^, CD8^+^CD45RA^+^, CD8^+^CD45RO^+^ and CD4^+^CD8^+^ T lymphocytes illustrate typical CD122 expression. (**B**) Percentages of CD45RA^+^ or CD45RO^+^ CD4^+^ and CD8^+^ T cells and CD4^+^CD8^+^ T lymphocytes expressing CD122 (left panel) as well as MFI for CD122 on positive cells (right panel) are shown as mean ± SEM (n = 7). Friedman test followed by Dunn’s multiple comparison test: CD4^+^CD8^+^ vs. CD4^+^CD45RA^+^ **p < 0.01. (**C**) PBMCs incubated in the absence or presence of different concentrations of IL-15 and IL-2 were stained for CD3, CD4, CD8, CD45RA, CD45RO, STAT3p and STAT5p. Representative contour plots of typical STAT3p and STAT5p expression in CD4^+^CD45RA^+^, CD4^+^CD45RO^+^, CD8^+^CD45RA^+^, CD8^+^CD45RO^+^ and CD4^+^CD8^+^ T lymphocytes either untreated (upper row) or stimulated with 5 ng/ml of IL-15 (middle row) or 1000 U/ml of IL-2 (bottom row) for 15 min are illustrated. (**D**) Percentage of each T cell subset expressing STAT5p following stimulation with different doses (0.2, 0.6, 1.0, 2.5 and 5.0 ng/ml) of IL-15 (left) or IL-2 (10, 50, 200, 500 and 1000 U/ml) (right) for 15 min. Mean ± SEM for IL-15 (n = 5) and for IL-2 (n = 7). Two way repeated measures ANOVA followed by Bonferroni post-tests comparing CD4^+^CD8^+^ subset to all others subsets *p < 0.05, **p < 0.01, ***p < 0.001.

Our data strongly suggest that CD4^+^CD8^+^ T lymphocytes display several characteristics of memory T lymphocytes. Therefore, we sought to determine whether these cells respond to key cytokines shaping T cell homeostasis, activation and survival. Amongst the cytokines acting on T lymphocyte subsets, we investigated the impact of IL-2, IL-15 and IL-7, which share one receptor chain (CD132, the common gamma chain)^26^. We assessed the expression of CD122, which is required for both IL-2 and IL-15 signalling. We observed that a greater proportion of CD4^+^CD8^+^ T lymphocytes (45.5 ± 8%) expressed detectable levels of CD122 compared to CD45RA^+^CD4 (3.4 ± 0.8%, **p < 0.01), CD45RO^+^CD4 (15.7 ± 3%) and CD45RA^+^CD8 (11.1 ± 3%) counterparts, while this proportion was slightly lower than in CD45RO^+^CD8 T lymphocytes (58.6 ± 8.1%) (Fig. 5A,B left panel). The density of CD122 on T cells as assessed by median fluorescence intensity (MFI) was similar for all subsets (Fig. 5B, right panel). To compare the response of T cell subsets to IL-15 and IL-2, we assessed the phosphorylation of STAT3 and STAT5^39^, two mediators shown to be involved in these cytokines’ downstream signalling. PBMCs were stimulated for 15 min, corresponding to the peak of response (data not shown), and then analysed simultaneously for each T lymphocyte subset. A representative example of the detection of STAT5 and STAT3 phosphorylation is illustrated in Fig. 5C for each subset and quantification for multiple donors and different cytokine concentrations is shown in Fig. 5D. IL-15 and IL-2 did not trigger detectable or significant STAT3 phosphorylation for all doses tested and T cell subsets analysed (Fig. 5C). In contrast, both cytokines induced a rapid and significant increase in the percentage of T cells expressing phosphorylated STAT5 in all subsets (Fig. 5C). The proportion of CD4^+^CD8^+^ T lymphocytes expressing phosphorylated STAT5 in response to IL-15 at all concentrations was significantly greater (**p < 0.01) compared to all other T cell subsets tested (Fig. 5D, left panel). As expected, IL-15 induced phosphorylation of STAT5 in a greater proportion of memory CD4 (5 ng/ml: 17.7 ± 3.6%) and CD8 (5 ng/ml: 30.7 ± 4.3%) T cells compared to their naïve counterparts (CD4: 5 ng/ml: 8.6 ± 1.8%; CD8: 5 ng/ml: 22.6 ± 4.9%).

IL-2, which also signals through CD122, triggered STAT5 phosphorylation in a greater proportion of memory T cells compared to naïve CD4 and CD8 counterparts at all doses tested (Fig. 5D). Notably, a significantly greater proportion of CD4^+^CD8^+^ T lymphocytes (62.4 ± 4.6% at 1000 U/ml) responded to IL-2 compared to CD45RA^+^CD4 (16.2 ± 1.6%), CD45RA^+^ CD8 (38.6 ± 2.8%), and CD45RO^+^CD8 (49.2 ± 5.8%) T cells; the response of CD45RO-expressing CD4 (54.5 ± 3.6%) was similar to CD4^+^CD8^+^ T lymphocytes (Fig. 5D, right panel). Overall, IL-15 and IL-2 triggered STAT5 phosphorylation in a significantly greater proportion of CD4^+^CD8^+^ T lymphocytes compared to naïve CD4 and CD8 T cell subsets. Moreover, IL-15 induced STAT5 phosphorylation in a higher percentage of CD4^+^CD8^+^ T lymphocytes compared to memory CD4 and CD8 T cells.

**Figure 6:**
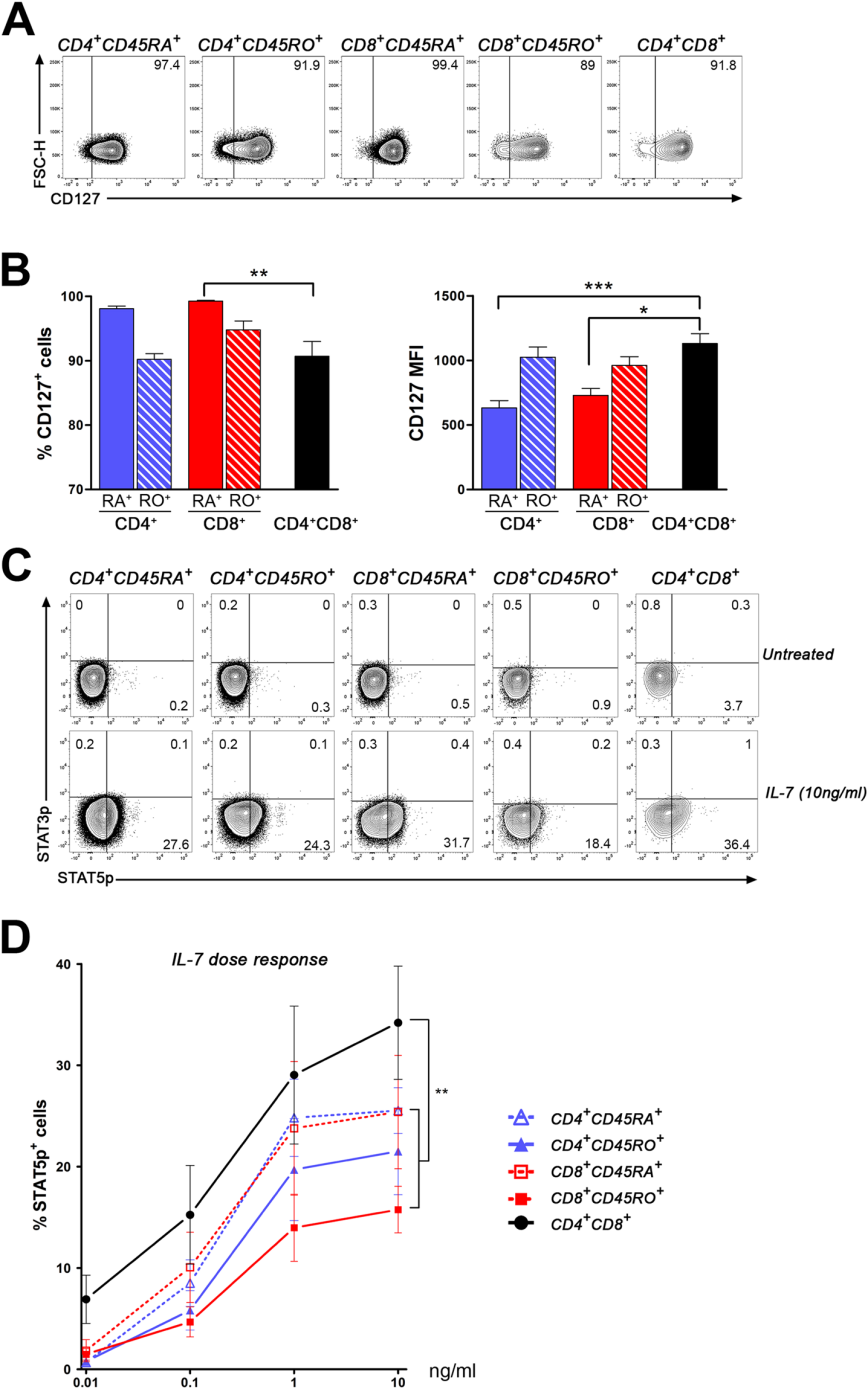
IL-7 triggers signalling in a greater proportion of CD4^+^CD8^+^ T lymphocytes than in other T cell subsets. CD4^+^CD8^+^ T lymphocytes were compared to other T cell subsets for their expression of CD127 the IL-7 specific receptor chain, as well as their capacity to respond to IL-7. (**A**) *Ex vivo* PBMCs were stained for CD3, CD14, CD4, CD8, CD45RA, CD45RO, and CD127. Representative contour plots of PBMCs from one donor gated on CD4^+^CD45RA^+^, CD4^+^CD45RO^+^, CD8^+^CD45RA^+^, CD8^+^CD45RO^+^ and CD4^+^CD8^+^ T lymphocytes illustrate typical CD127 expression. (**B**) Percentages of CD45RA^+^ or CD45RO^+^ CD4^+^ and CD8^+^ T cells and CD4^+^CD8^+^ T lymphocytes expressing CD127 (left panel) as well as MFI for CD127 on positive cells (right panel) are shown as mean ± SEM (n = 7). (**C**) PBMCs incubated in the absence or presence of different concentrations of IL-7 were stained for CD3, CD4, CD8, CCR7, CD45RA, CD45RO, STAT3p and STAT5p. Representative contour plots of typical STAT3p and STAT5p expression in CD4^+^CD45RA^+^, CD4^+^CD45RO^+^, CD8^+^CD45RA^+^, CD8^+^CD45RO^+^ and CD4^+^CD8^+^ T lymphocytes either untreated (upper row) or stimulated with 10ng/ml of IL-7 (bottom row) for 15 min are illustrated. (**D**) Percentage of each T cell subset expressing STAT5p following stimulation with different doses (0.001, 0.01, 0.1, 1.0 and 10 ng/ml) of IL-7 for 15 min is shown as mean ± SEM (n = 6). Two way repeated measures ANOVA followed by Bonferroni post-tests comparing CD4^+^CD8^+^ subset to all others subsets **p < 0.01.

We also investigated the impact of IL-7, another key cytokine for the homeostasis and survival of naïve and memory T lymphocytes^40^. First, we assessed the expression of CD127, which is required for IL-7 signalling. We observed that a similar elevated proportion of CD4^+^CD8^+^ T lymphocytes expressed CD127 (90.7 ± 2.3%) compared to CD45RO^+^CD4 (90.2 ± 0.9%) and CD8 (94.8 ± 1.4%) but this proportion was lower than in CD45RA^+^CD4 (98.1 ± 0.4%) and CD8 T lymphocytes (99.2 ± 0.1%, **p < 0.01 compared to CD4^+^CD8^+^ T cells) (Fig. 6A,B, left panel). The average CD127 density on CD4^+^CD8^+^ T lymphocytes (MFI: 1130 ± 77) was significantly greater than on CD45RA-expressing CD4 (633 ± 56; ***p < 0.001) and CD8 T cells (729 ± 53; *p < 0.05) but similar to the levels detected on CD45RO-expressing CD4 (1024 ± 80) and CD8 (962 ± 67) T cells (Fig. 6B, right panel).

We also measured T cell responses to IL-7 using STAT3 and STAT5 phosphorylation, as these molecules are also involved in IL-7-mediated signalling^40^. A representative example of the detection of STAT5 and STAT3 phosphorylation is illustrated in Fig. 6C. Similar to what we observed for IL-15 and IL-2, we did not detect STAT3 phosphorylation following IL-7 stimulation in T cell subsets (Fig. 6C). In contrast, STAT5 phosphorylation was quickly triggered in all T cell subsets (Fig. 6C). The proportion of CD4^+^CD8^+^ T lymphocytes expressing phosphorylated STAT5 in response to IL-7 (10 ng/ml: 34.2 ± 5.6%) was significantly higher than the other T cell subsets tested: CD4^+^CD45RA^+^ (25.4 ± 5.6%), CD4^+^CD45RO^+^ (21.5 + 4.3%), CD8^+^CD45RA^+^ (25.4 + 5.6%), CD8^+^CD45RO^+^ (15.8 + 2.3%) (Fig. 6D).

Our results demonstrate that CD4^+^CD8^+^ T lymphocytes exhibit an enhanced capacity to trigger STAT5 phosphorylation in response to IL-15, IL-2 and IL-7, three members of the type I common γ chain (CD132) cytokine family^41^, compared to naïve T cell subsets and a greater enhanced response to IL-15 and IL-7 in comparison to memory T cell subsets.

## Discussion

Several lines of evidence support the existence of CD4^+^CD8^+^ T lymphocytes in the periphery as mature T lymphocytes^1^. Although these cells are found in numerous species and related to different disease conditions, the phenotype and the functions of these T lymphocytes are still incompletely resolved. In this study, we demonstrated that CD4^+^CD8^+^ T lymphocytes from healthy donors exhibit *i)*multiple phenotypic markers associated with memory T lymphocytes, *ii)*an increased capacity to produce cytokines and lytic enzymes, and *iii)*an elevated proportion of cells responding to key cytokines implicated in T cell survival, homeostasis and activation.

Very few studies^7, 9^ have previously assessed the phenotype of human CD4^+^CD8^+^ T lymphocytes *ex vivo* in the absence of any *in vitro* culture. We compared the expression of representative molecules such as chemokine receptors (CCR7, CXCR3, CCR6), co-activating receptors (NKG2D, CD27, CD28), differentiation stage markers (CD45RA, CD45RO, CD57, CD95) and cytokine receptors (IL-18 Rα, CD122, CD127) on *ex vivo* CD4^+^CD8^+^ T lymphocytes to other T cell subsets. Other groups divided CD4^+^CD8^+^ T cells into smaller subsets based on different levels of CD4, CD8, or chains of CD8^37, 38^. We observed as previously reported that the vast majority of CD4^+^CD8^+^ T cells in human blood express the CD8αβ heterodimer while a very small number of cells carried CD8α in the absence of CD8β (data not shown). In contrast, the gut derived cells have been shown to carry the CD8αα homodimer^28^. We compared CD4^lo^CD8^hi^, CD4^hi^CD8^lo^ and CD4^hi^CD8^lo^ subsets for the production of IFNγ and we did not see any difference between these subsets (Fig. 3).

We observed that CD4^+^CD8^+^ T lymphocytes could easily be distinguished from naïve CD4 and CD8 T lymphocytes based on the proportions of these cells lacking CCR7, CD27, and CD28, but expressing CD57, CD95, CD45RO, CCR6, CXCR3, IL18Rα, and CD122 (Figs 1, 2, 3 and 5). Others similarly detected an increased proportion of CD45RO^+^CCR7^−^ and CXCR3^+^ cells in CD4^+^CD8^+^ T lymphocytes compared to CD4 and CD8 T cells^7, 42^. Nascimbeni and colleagues also showed an elevated proportion of CD27^−^, CD28^−^ or CD57^+^ human CD4^+^CD8^+^ T cells compared to CD4 and CD8 T cells without discriminating for specific subsets^7^. Using the CCR7, CD45RO, CD28 and CD95 expression profile (Fig. 2B) to discriminate differentiation stages (Tn, Tscm, Tcm, Ttm, Tem, Tte)^33^, we observed that more than 75% of CD4^+^CD8^+^ T cells did not display a naïve profile. Indeed, over half of the CD4^+^CD8^+^ T lymphocytes exhibited the characteristics of Tcm, whereas very few of these cells expressed either the Tscm or Tem profile. We observed that more than half of CD4^+^CD8^+^ T lymphocytes expressed NKG2D, a co-activating receptor mainly present on CD8 T lymphocytes (Fig. 1B,C). Others reported NKG2D expression on CD4^+^CD8^+^ T cells after *in vitro* activation of murine CD4 T cells or expansion of tumour infiltrating T lymphocytes^9, 43^. The expression profile of CD4^+^CD8^+^ T lymphocytes for some molecules (e.g. NKG2D, CXCR3) was more similar to CD8 than CD4 T cells, whereas the proportion of these cells expressing other markers (CD57, CD27, CD28) was in between those observed in central memory and effector memory subsets. We can conclude that, as for CD4 and CD8 T cells, CD4^+^CD8^+^ T cells represent a heterogeneous T cell population. Overall, our *ex vivo* T cell phenotyping supports the notion that CD4^+^CD8^+^ T lymphocytes exhibit characteristics of memory T cells from both CD4 and CD8 subsets.

As most CD4^+^CD8^+^ T lymphocytes exhibit characteristics of memory T cells, we evaluated whether these cells display properties (chemokine/cytokine receptor and production of cytokines and lytic enzymes) associated with specific T cell polarization (Figs 3 and 4). We observed that similar proportions of CCR6^−^CXCR3^+^ Th1, CCR6^+^CXCR3^−^ Th17 and CCR6^+^CXCR3^+^ Th1/Th17 cells were present within the peripheral CD4^+^CD8^+^ T lymphocytes compared to central memory and effector memory CD4 T cells (Fig. 3A). In contrast, significantly reduced proportions of CCR6^−^IL-18Rα^+^ Tc1 cells and augmented CCR6^+^IL-18R^−^ Tc17 were detected compared to effector memory CD8 T cells (Fig. 3B). Notably, an important percentage of CD4^+^CD8^+^ T lymphocytes demonstrated the capacity to produce IFNγ, TNFα, and lytic enzymes (GrB and perforin); these effector molecules are associated with Th1/Tc1 cells (Figs 3 and 4). Suni and colleagues showed that in response to CMV and HIV-1, CD4^+^CD8^+^ T lymphocytes have enhanced cytolytic functions compared to their CD4 counterparts^13^. Previous reports also identified a population of intestinal intraepithelial CD4^+^CD8^+^ T lymphocytes producing IFNγ^16, 43^. On the other hand, a greater proportion of CD4^+^CD8^+^ T cells could produce IL-4 following a short *in vitro* stimulation (Fig. 3C,D) compared to other T cells similar to what has been previously published by others^28^. Elevated proportions of IL-4-producing CD4^+^CD8^+^ T cells have been detected in patients suffering from rheumatoid arthritis or colorectal or breast cancers compared to controls^9, 10, 17^. However, some studies evaluated T cells after an extensive *in vitro* culture step^9^. The similar small percentage (1.9%) of CD4^+^CD8^+^ T cells and CD4 T cells producing IL-17A we observed was higher than what Quandt and colleagues reported^10^. Such differences could be due to the fact that we used brefeldin A (BFA) instead of monensin to block cytokine secretion. Our results suggest that CD4^+^CD8^+^ T cells contain a large proportion of cells exhibiting Th1 properties (CXCR3^+^, IFNγ, lytic enzymes), but also small proportions of other subsets (e.g. IL17A- or IL-4-producers).

IL-2, IL-15 and IL-7 play essential roles in the homeostasis, survival and activation of T lymphocytes^26^. Using STAT5 phosphorylation as a readout of these cytokines triggering effects in T cells^44, 45^, we observed a significantly enhanced capacity of CD4^+^CD8^+^ T lymphocytes to respond to all three cytokines compared to other T cell subsets (Figs 5 and 6). These enhanced responses could not be solely explained by receptor chain expression. Indeed, the proportion of CD4^+^CD8^+^ T lymphocytes expressing detectable levels of CD122, which is required for both IL-2 and IL-15 signalling, was greater compared to CD4 T cells and naïve CD8 T cells, but similar to memory CD8 T cells (Fig. 5A,B). Moreover, similar proportions of memory CD4, memory CD8 and CD4^+^CD8^+^ T lymphocytes expressed CD127, the IL-7Rα chain. Nevertheless, we observed a significantly greater proportion of CD4^+^CD8^+^ T lymphocytes in which STAT5 phosphorylation was triggered in response to IL-15 and IL-7 compared to all CD4^+^ and CD8^+^ T lymphocyte subsets (Figs 5D and 6D). Other studies reported IL-2- or IL-15-triggered STAT3 phosphorylation in pre-activated lymphocytes^44, 45^; we speculate that this pre-activation step (phytohemagglutinin or concavalin A) modified cell status and could explain why we did not observed any STAT3 phosphorylation in freshly isolated PBMCs (Figs 5C and 6C). We can also rule out a technical problem as we routinely detect phosphorylated STAT3 in human T cells in response to IL-27^46^. We can speculate that CD4^+^CD8^+^ T lymphocytes could have a survival advantage over other T cell subsets. Indeed, IL-15 can inhibit IL-2-activation induced cell death of T cells^25^, increase anti-apoptotic protein levels (e.g. Bcl-2) and the anti-oxidant capacity of T cells^47, 48^. IL-7 plays an essential role in lymphocyte homeostasis as it favours T cell proliferation while maintaining these cells in their maturation stage^49^. Moreover, CD4^+^CD8^+^ T cells have been detected in multiple human tissues (liver, lymph nodes, colon, skin, etc.)^1^. Notably, IL-15 favours the migration of memory CD8 T cells to inflamed tissues^50^; whether this cytokine has the same impact on CD4^+^CD8^+^ T cells remains to be investigated. We also measured STAT phosphorylation in response to other cytokines (IL-27 and IL-9), but CD4^+^CD8^+^ T lymphocytes did not display enhanced response to these cytokines compared to CD4^+^ and CD8^+^ T lymphocytes (data not shown).

Several publications have investigated CD4^+^CD8^+^ T lymphocytes and reported increased frequency of these cells with aging and in the context of infectious and autoimmune diseases, as well as cancer^1, 5, 7, 14^. Given the memory phenotype, cytotoxic profile, and enhanced capacity to respond to key survival cytokines that we observed *ex vivo* for CD4^+^CD8^+^ T lymphocytes, we can speculate that multiple immune activation circumstances can favour the presence of these cells in the periphery and potentially in organs. Additional studies are deemed essential to better understand the mechanisms controlling the co-expression of CD4 and CD8 molecules and whether such co-expression confers any functional advantage.

## Materials and Methods

### Cell isolation and culture

Written informed consent was obtained from healthy adult donors in accordance with the local ethical committee and these studies were approved by the Centre Hospitalier de l’Université de Montréal ethical boards (BH 07.001 and CE 13.040). Donors were between 21 and 60 years old (mean 35 ± 1.8 years old) and included 47% women and 53% men. All methods were performed in accordance with the relevant guidelines and regulations. Peripheral blood mononuclear cells (PBMCs) were isolated from blood samples collected in EDTA-coated tubes (BD Biosciences, Mississauga ON Canada) using Ficoll density gradient as previously described^46, 51, 52^. To assess cytokine production, PBMCs were stimulated with phorbol 12-myristate 13-acetate (20 ng/ml) (Sigma-Aldrich, Oakville, ON, Canada) and ionomycin (500 ng/ml) (Sigma-Aldrich) in the presence of brefeldin A (5 ug/ml) (Sigma-Aldrich) for 5 hours before flow cytometry analysis as previously described^51^. To assess degranulation, PBMCs were stimulated with phorbol 12-myristate 13-acetate (20 ng/ml) and ionomycin (500 ng/ml) in the presence of monensin (1 mM) (Sigma-Aldrich) and CD107a antibody (BD Biosciences) or corresponding isotype for 5 hours. Cells were then harvested and stained for others markers before flow cytometry analysis. To determine cytokine-triggered STAT signalling, PBMCs were rested at 37 °C in Iscove’s Modified Dulbecco’s Medium (Life Technologies Thermo Fisher Scientific, Burlington, ON, Canada) without serum for 1 hour and then activated with human recombinant IL-2 (Roche, Nutley, NJ, USA), IL-15 (R&D Systems, distributed by Cedarlane Oakville, ON, Canada) or IL-7 (Peprotech, distributed by Cedarlane Oakville, ON, Canada) for 15 minutes, quickly put on ice and finally processed for flow cytometry analysis.

### Flow cytometry

PBMCs were stained for surface markers and/or intracellular cytokines as previously described^46, 51, 52^. Briefly, cells were blocked for 15 min at 4 °C with normal mouse immunoglobulins (mIgG) (6ug mIgG/million cells) (Invitrogen, ThermoFisher Scientific) and then incubated with fluorochrome-labelled antibodies targeting surface antigen (see antibodies Table 1) for 30 min at 4 °C. To exclude dead cells, LIVE/DEAD Fixable Aqua Dead Cell Stain (Molecular Probes™, ThermoFisher Scientific) was added simultaneously to the surface staining step. To assess phosphorylation of STATs, PBMCs were first surface stained, then fixed with 1.5% paraformaldehyde, and finally permeabilised with PermBuffer III (BD Biosciences) for 25 min on ice according to the manufacturer’s instructions. Cells were acquired on a LSRII flow cytometer (BD Biosciences) and analysed using FlowJo software (Treestar, Ashland, OR, USA). To ensure stringent single-cell gating, doublets were excluded using SSC and FSC Height and Width as recommended by the Flow Cytometry Network (www.thefcn.org); single events were first gated on the SSC-H vs. SSC-W and then on the FSC-H vs. FSC-W dot plots. Appropriate isotype controls were used in all steps. Staining specificity was confirmed using fluorescence minus one (FMO, all antibodies minus one). The median fluorescence intensity (MFI) was calculated by subtracting the fluorescence of the isotype from that of the stain.

**Table 1:** List of antibodies used.

Targeted human antigen-fluorochrome	Clone	Source
CD3-Alexa Fluor® 700	UCHT1	BD Biosciences
CD4-Pacific Blue™	RPA-T4	BD Biosciences
CD4- BV786	SK23	BD Biosciences
CD4-APC	RPA-T4	BD Biosciences
CD8-APC-Cy™7	SK1	BD Biosciences
CD8-Pacific Blue™	RPA-T8	BD Biosciences
CD8-PE	HIT8a	BD Biosciences
CD14-APC	M5E2	BD Biosciences
CD14-APC-H7	M5E2	BD Biosciences
CD27-Brillant Violet 785™	O323	BioLegend
CD28-Biotin	CD28.2	BioLegend
CD28-PerCP-Cy™5.5	L293	BD Biosciences
CD45RA-FITC	HI100	BD Biosciences
CD45RO-PerCP-Cy™5.5	UCHL1	BD Biosciences or BioLegend
CD45RO-PE	UCHL1	BD Biosciences
CD56-PE-Cy™7	B159	BD Biosciences
CD57-APC	NK-1	BD Biosciences
CD95-FITC	DX2	BD Biosciences
CD107a-PE	H4A3	BD Biosciences
CD122-PE	Mik-β2	BD Biosciences
CD127- Alexa Fluor® 488	A019D5	BD Biosciences
TCRαβ-FITC	WT31	eBioscience
TCRγδ-Biotin	B1	BD Biosciences
iαTCR-PE	6B11	BioLegend
CCR6-PE-CF594	11A9	BD Biosciences
CCR7-PE-Cy™7	3D12	BD Biosciences
CCR7-PE-Cy™7	G043H7	BioLegend
CXCR3-PE	49801	R&D Systems
CXCR3-Brillant Violet 421™	G025H7	BioLegend
IL-18Rα-Biotin	H44	BioLegend
PD1-PE	J105	eBioscience
NKG2D-APC	1D11	BioLegend
IFNγ-Alexa Fluor® 488	45.B3	BD Biosciences
IL-2-PE	5344.111	BD Biosciences
IL-4-PE	8D4-8	BD Biosciences
IL-17A-EFluor® 660	64CAP17	eBioscience
TNFα- PE-Cy™7	MAb11	BD Biosciences
Granzyme B-Alexa Fluor® 647	GB11	BD Biosciences
Perforin-PE	B-D48	Abcam
STAT3p-Alexa Fluor® 647	4/p-STAT3	BD Biosciences
STAT5p-PE	47	BD Biosciences
Streptavidin-BV605	N/A	BD Biosciences
Streptavidin-APC	N/A	BD Biosciences

### Statistical analysis

Data analysis was performed using Prism 5.0 software (GraphPad, La Jolla, CA, USA). Results are represented as mean ± SEM. When data passed the D’Agostino & Pearson omnibus normality test, ANOVA followed by Dunnett’s multiple comparison test was used. When data did not pass the normality test, the Friedman test followed by a Dunn’s multiple comparison test was used. Two way repeated measures ANOVA followed by Bonferroni post-test were used for analysis of dose responses to cytokines. Values were considered statistically significant when probability (P) values were equal or below 0.05 (*), 0.01 (**), or 0.001 (***).
